# Only a Burden for Females in Math? Gender and Domain Differences in the Relation Between Adolescents’ Fixed Mindsets and Motivation

**DOI:** 10.1007/s10964-020-01345-4

**Published:** 2020-11-10

**Authors:** Anke Heyder, Anne F. Weidinger, Ricarda Steinmayr

**Affiliations:** Department of Psychology, Technical University Dortmund, Emil-Figge-Str. 50, D- 44227 Dortmund, Germany

**Keywords:** Mindsets, Gender differences, Ability self-concept, Intrinsic motivation, Math

## Abstract

Gendered occupational and educational choices have often been traced back to gender differences in students’ domain-specific ability self-concept and intrinsic motivation. This study explored the role of believing in an “innate” math or language arts ability (i.e., having a fixed mindset) for gender differences in students’ ability self-concept and intrinsic motivation in 423 female (49%) and 447 male (51%) tenth graders from Germany (age *M* = 16.09 years, SD = 0.68, range: 14–18 years). In line with math-male stereotypes, believing in “innate” math ability was associated with lower ability self-concept and intrinsic motivation in female but not male students. In language arts, students’ mindsets were unrelated to their motivation. The results suggest that a fixed mindset presents an additional burden for female students in math, but not for male or female students in language arts.

## Introduction

Choosing a career is an important decision to make for adolescents. Although almost all occupations are open to both women and men, students’ decisions are still strongly influenced by their gender. For instance, whereas teachers, secretaries, or registered nurses have a women’s share larger than 87%, less than 10% of the electricians, mechanics, and computer network architects are female (US Department of Labor 2017). In science, technology, engineering, and math (STEM), women make up only 28% of the workforce overall (National Science Board 2018). These gendered choices and particularly the underrepresentation of women in STEM have far-reaching effects at the expense of women. Math-related or STEM-related careers are more prestigious than other careers (Watt et al. 2012) and better paid in the US (US Bureau of Labor Statistics 2020) as well as in European countries such as Germany (Federal Employment Agency 2020). What explains the underrepresentation of women in STEM in college and workforce? Average achievement differences at the expense of female students have been ruled out as the primary explaining factor (e.g., Wang and Degol 2016), shifting the focus to adolescents’ *motivation* to pursue prestigious STEM-careers (for a recent review, see also Master and Meltzoff 2020). This study therefore aimed to explain gender differences in two powerful motivational predictors of students’ academic and occupational choices, namely their ability self-concept and intrinsic motivation in an academic domain (e.g., Eccles [Parsons] et al. 1983). More precisely, it examined how adolescents’ mindsets, that is their beliefs about whether abilities are fixed or malleable (e.g., Dweck and Yeager 2019), are related to their ability self-concept and intrinsic motivation, and whether these relations differ depending on students’ gender. Extending prior research, this study relied on a German sample and focused not only on the relations in math, a highly-studied, prestigious and male-stereotyped domain, but also in language arts, a less-studied, less prestigious and female-stereotyped domain.

### Gender Differences in Math and Language Motivation

According to the expectancy-value theory (EVT; Eccles [Parsons] et al. 1983), students’ expectancies and values in academic domains—like their ability self-concepts and intrinsic motivation in math and language arts—determine their choices and persistence. A variety of empirical studies has found that girls’ ability self-concept and intrinsic motivation in math were significantly lower than boys’ (e.g., Watt 2006) although gender differences in average math achievement (e.g., Else-Quest et al. 2010) or the perceived usefulness of math (e.g., Watt 2006) were relatively weak or nonexistent. In the verbal domain, by contrast, boys reported a lower ability self-concept (e.g., Heyder et al. 2017) and lower intrinsic motivation (e.g., Durik et al. 2006) than girls which is in line with their lower reading competencies (e.g., Reilly et al. 2019) or language grades (e.g., Heyder et al. 2017). Supporting EVT, these gender differences in domain-specific motivation evolved into substantial gender differences in career aspiration and choices, like course-choices in high school (e.g., Wang and Degol 2013), career goals, and career attainment later on (e.g., Lauermann et al. 2017).

Since gender differences in motivation can have such far-reaching consequences for students’ careers, it is worthwhile to investigate and to better understand factors that shape these gender differences. According to EVT, differences between boys’ and girls’ motivation are, amongst others, determined by their perception of gender stereotypes, that is, beliefs about the attributes of females and males as a social group, and beliefs about the nature of abilities (see Wigfield et al. 2016), in what follows called mindsets. Also the socio-cognitive model (e.g., Dweck and Leggett 1988) assumes students’ mindsets to affect students’ subsequent motivation. This study focused on students’ mindsets in math and language arts and explored their role in gender differences in students’ motivation in these domains.

### General and Domain-Specific Mindsets

Mindsets can be defined as a person’s subjective beliefs about whether a particular attribute, such as intelligence or math ability, is fixed or can be shaped and developed (e.g., Dweck and Yeager 2019). The first belief describes a fixed mindset whereas the second belief describes a growth mindset. Unlike a fixed mindset, having a growth mindset about one’s abilities is theorized to foster students’ motivation and achievement, in particular for struggling students. When confronted with difficulties, students’ mindsets affect their reaction (e.g., Dweck and Leggett 1988). That is, if a student believes in a fixed ability, experiencing that there is something she or he does not succeed in decreases the student’s motivation, elicits low-ability attributions, and undermines her or his achievement in the long run. By contrast, if a student believes in a malleable ability, experiencing failure only means that there is something she or he has not succeeded in *yet*. A growth mindset thus protects students’ motivation and self-evaluation because there is the option to improve, learn, and grow, and thus fosters their achievement. Several empirical studies inducing or fostering domain-unspecific growth mindsets of intelligence in students have supported these predictions (e.g., Paunesku et al. 2015). Furthermore, believing that human attributes are rather malleable than fixed was found to positively correlate with learning goals, mastery-oriented strategies, expectations (Burnette et al. 2013) and achievement (Sisk et al. 2018) in meta-analyses. Even though the effect sizes were not large, these results suggest that students’ mindsets matter for students’ motivation and achievement.

Recently, the question whether people perceive abilities in different academic domains as more or less malleable has gained more attention. Here, research suggests that adolescents and adults believe more strongly in an “innate” ability that is required to be successful in math than in language arts (e.g., Gunderson et al. 2017). Basically, they hold stronger fixed mindsets of math ability than of abilities in other domains. Moreover, students with low math competencies (Seo et al. 2019), low prior math achievement (Degol et al. 2018), and high socioeconomic status (e.g., Seo et al. 2019) as well as White US students compared to Hispanic or Asian US students (Hwang et al. 2019) were more likely to endorse fixed mindsets of math ability. These studies suggest that students’ prior achievement, domain-specific competencies, socioeconomic and cultural background might be confounded with their mindsets in math, and should therefore be statistically controlled for when investigating students’ mindsets in math. This study aims to explore whether domain-specific mindsets play out differently for female and male students’ motivation.

### Gender-Specific Effects of Students’ Mindsets

Current gender stereotypes ascribe higher “innate” math ability to male than to female individuals (e.g., Steffens and Jelenec 2011). For the verbal domain in contrast, female persons are believed to possess more “innate” ability than male persons. These gender stereotypes are still widespread and even hidden in seemingly gender-equal statements such as “girls do as good in math as boys” or “boys’ verbal ability is as good as girls’” (Chestnut and Markman 2018). Gender stereotypes about students’ ability are crucial to understand the effects of students’ domain-specific mindsets for female and male students’ motivation in math and language. Because female students are stereotyped as lacking the required “innate” math ability compared to male students, having a fixed mindset of math ability should be more detrimental for female than male students’ motivation. The stereotype that male students have higher “innate” math ability might even protect them from the negative effects of having a fixed mindset in math. For the verbal domain, opposite relations are expected, that is, more detrimental effects of a fixed mindset for male than female students’ motivation.

So far only two studies have directly addressed the question of gender-specific relations between students’ mindsets and motivation. They were both located in the US and focused on math. In a sample of students from Grade 9 to 12, growth mindsets in math were positively related to concurrent expectancies of success only among female but not among male students, whereas growth mindsets were positively related to concurrent math values, irrespective of students’ gender (Degol et al. 2018). In the Education Longitudinal Study 2002 data, there was a positive association between students’ math growth mindset in Grade 10 and their math ability self-concept in Grade 12 which did not significantly differ depending on students’ gender or ethnicity (Seo et al. 2019). Due to the differences in students’ age, measures, and analyses, the findings of these two studies are hard to integrate and call for further research on this topic. Several studies provide additional, indirect evidence for gender-specific effects of students’ mindsets. For instance, the degree to which an academic domain is perceived as requiring “innate” ability to succeed was negatively related to the proportion of female PhD in this academic domain (Leslie, Cimpian et al. 2015). Domains such as math that were perceived as requiring more “innate” ability than others were characterized by a lower portion of women, suggesting that such beliefs represent a barrier particularly for women (see also Meyer et al. 2015). Moreover, perceiving faculty members as believing that not everyone has the ability to succeed in STEM was negatively related to women’s—but not men’s—sense of belonging in STEM and produced a gender gap in students’ sense of belonging (Rattan et al. 2018; Studies 1 and 6). Finally, fostering a growth mindset of math ability increased only female students’ achievement in standardized math tests with the effect that prior gender differences in math achievement disappeared (Good et al. 2003).

Taken together, empirical findings on whether fixed mindsets in math matter more for female students stereotyped as lacking “innate” math ability than for male students stereotyped as possessing “innate” math ability are mixed, calling for further research. Moreover, several other open questions remain. First, whether students’ mindset in math matters more, not only for female than male students’ ability self-concept, but also for their intrinsic motivation has not been studied yet. This is important as female students consistently report lower intrinsic motivation in math than male students but not necessarily lower scores in other value components (e.g., Gaspard et al. 2015). Furthermore, the interaction between ability self-concept and intrinsic motivation (and not between ability self-concept and utility value) has been found to predict students’ occupational choices above and beyond their individual effects (e.g., Lauermann et al. 2017). Second, all known studies on gender-specific mindset effects studied the effects in math, an academic domain stereotyped as male. Thus, it is unclear, whether opposite relations occur in other domains stereotyped as female, such as the verbal domain. However, a better understanding of factors that lead to the gender-stereotypical choices of these domains is important in order to comprehensively counsel and support adolescents in making the individually right decision about their careers. Third, all known studies on the gendered effects of students’ mindsets relied on data from the US. Thus, research from other countries is needed to estimate the robustness and generalizability of prior findings across countries or cultures.

## Current Study

The lower math ability self-concept and intrinsic motivation in math of female than male adolescents are two important precursors of women’s underrepresentation in prestigious STEM occupations. Prior research and theory suggested that a fixed mindset of math ability may represent an additional burden for female adolescents in math, but findings vary between constructs and measures. Furthermore, it is unclear whether this potential burden tackles both female adolescents’ ability self-concept and intrinsic motivation in math and holds for samples from outside the US, and whether opposite relations exist in academic domains dominated by women, like verbal domains. To address this research gap, this study analyzed the relation between adolescents’ mindset, ability self-concept, and intrinsic motivation in two gender-stereotyped and opposed domains, math and German language art, in Germany. Because of the gender stereotyping of math as a “male” and language arts as a “female” domain, gender is expected to moderate the relation between students’ mindsets and their motivation. First, a fixed mindset of math ability should be negatively related to female students’ math ability self-concept (H1a) and their intrinsic motivation in math (H1b), but not necessarily to male students’ because female students are at higher risk to be stereotyped as lacking “innate” math ability. Second, the opposite pattern is expected for language arts. That is, a fixed mindset of language arts ability should be negatively related to male students’ ability self-concept (H2a) and intrinsic motivation (H2b) in this domain, but not necessarily to female students’ because female students are stereotyped as having higher “innate” talent for language arts than male students. All hypotheses will be tested with and without including students’ prior achievement, domain-specific competencies, socioeconomic and cultural background as control variables.

## Methods

### Sample and Procedure

This study draws on student data from the FA(IR)BULOUS project, a two-wave longitudinal German research project on social inequality in school transitions (Steinmayr et al. 2017). Specifically, it re-analyzed the Wave 2 secondary-school data from this project that was collected in spring 2017 in North Rhine-Westphalia, Germany. Wave 2 was in focus because only here students’ mindsets were assessed.

The Wave 2 sample consisted of 877 tenth graders, nested in 51 classes. Tenth grade is crucial because in this grade, many students are faced with the decision whether to enter the labor market or continue schooling, and consequently, which occupation to choose or which courses to enroll in. Approximately half of the sample (48.2%, *n* = 423) was female, the other half male (51.0%, *n* = 447; seven missing statements). Students were *M* = 16.09 years old (SD = 0.68; range: 14–18 years). The majority (82.0%, *n* = 719) reported to speak German at home, 152 reported another language than German (17.3%, six missing statements). The German education system is characterized by an early formal differentiation between different school types (for more information, see LeTendre et al. 2003). In this sample, 30.1% of students (*n* = 264) attended comprehensive schools (Gesamtschule), 53.0% (*n* = 465) attended intermediate track schools (Realschule), and 16.9% (*n* = 148) attended schools of the lowest track (Hauptschule)^1^. The sample was roughly representative for the student population attending the three types of secondary schools in 2017 in North-Rhine Westphalia with respect to their distribution across the three school types, gender ratio and the language they spoke at home (MSW 2017; see Table S1 in the online supplement). The sample of students from medium- and low-track schools that do not prepare students for higher education represents an understudied group compared with academic-track students from Germany or students from the US. At the same time, more than half of the persons working in STEM professions in Germany do not have a university degree (Federal Employment Agency 2020) and fixed mindsets might be especially detrimental to low-track students’ motivation because of the low-ability-stigma associated with the low–track schools in Germany (Knigge and Hannover 2011).

The data were collected during regular class hours by trained research assistants in the second term of tenth grade (April–June 2017). First, students answered questions on their gender, age, and social background. Then they completed questionnaires on their motivation and mindsets as well as on other variables not of interest for this study, and they worked on standardized math competence and reading comprehension tests. In compliance with established ethical principles, students’ participation was voluntary and parents’ informed consent was obtained before participation. Participation rate was ~70%. Students who did not participate missed school on the day of testing due to reasons not related to the project such as illness. The responsible school administrations approved the study design and the data collection procedure beforehand.

### Measures

#### Ability self-concept

Students’ ability self-concepts in math and German language arts were each assessed with four items taken from the German Scales for the Assessment of School Related Competence-Beliefs (Schöne et al. 2002). A sample item is “I am good in math/in German” (for all items, see Spinath and Steinmayr [2012], p. 1148). Students answered all items on a scale from 1 (*totally disagree*) to 5 (*totally agree*). The internal consistency of the scale was high in math (Cronbach’s α = 0.93) and German language arts (Cronbach’s α = 0.89).

#### Intrinsic motivation

Students’ intrinsic motivation in math and German language arts were assessed with four items each (see also Heyder et al. 2020). The first three items were the enjoyment and interest items from the Program for International Student Assessment (PISA; Prenzel et al. 2013) “I am interested in the things I learn in math/German”, “I look forward to my math lessons/German lessons”, and “I do math/German because I enjoy it.” Item 4 (“I enjoy doing things in math/German”) stemmed from the German Scale for the Assessment of School Related Values (Steinmayr and Spinath 2010) that is based on the EVT (e.g., Eccles [Parsons] et al. 1983). Students answered all items on a scale from 1 (*totally disagree*) to 5 (*totally agree*). The internal consistency of the scale was high in math (Cronbach’s α = 0.91) and German language arts (Cronbach’s α = 0.89).

#### Fixed mindset

Per domain, students indicated on a 7-point scale from 1 (*strongly disagree*) to 7 (*strongly agree*), whether they agreed with two positively-worded fixed mindset items (“Being among the best in math/German requires a special aptitude that just cannot be taught”; “If you want to succeed in math/German, hard work alone just won’t cut it; you need to have an innate gift or talent”) and one negatively-worded fixed mindset item (“When it comes to math/German, the most important factors for success are motivation and sustained effort; raw ability is secondary.”). The items were used before in US American (Leslie, Cimpian et al. 2015) and German (Heyder et al. 2020) adult samples and minimally adapted for school students. Preliminary analyses revealed that in this sample the reversed coded third item was only weakly correlated with the two positively worded items in math (0.17 ≤ *r* ≤ 0.30) as well as German (*r* = 0.17), and internal consistencies were low (α = 0.61 math, α = 0.60 German language arts). In order to obtain a reliable measure of students’ mindset, the negatively-worded item was thus excluded, resulting in a pure fixed mindset measure with good internal consistency (Cronbach’s α = 0.79 in both domains).

#### Prior achievement

Schools provided students’ last report card grades in math and German that indicated their cumulative prior achievement in the first term of Grade 10. In Germany, report card grades range from 1 (*excellent performance*) to 6 (*insufficient performance*).

#### Math competencies

Students’ math competencies were assessed by the KRW test (*K*onventions- und *R*egel*w*issenstest), a supplemental test of the German mathematic test for 9th grade (DEMAT 9; Schmidt et al. 2013). The KRW test consists of 50 typical math operations to be solved in three and a half minutes (*r*_*tt*_ = 0.77 for 8 weeks).

#### Reading comprehension

Students’ reading comprehension was assessed by the LGV test (*L*ese*g*eschwindigkeits- und *V*erständnis*t*est; Schneider et al. 2007). Here, students’ task was to read a text with 1727 German words for four minutes and to select from three alternatives in 23 sentences the word that fits best into the text context (*r*_*tt*_ = 0.87 for 6 weeks).

#### Demographic variables

Students reported their gender, the language spoken at home (0 = *German*, 1 = *other language*), that is frequently used as an indicator of migration background in Germany (e.g., see Kigel et al. 2015), and, as in large scale studies such as PISA, the number of books at home as an indicator of students’ home resources for learning (*1* = *0–10 books, 2* = *11–50 books, 3* = *51–100 books, 4* = *101–250 books, 5* = *251–500 books, 6* = *>500 books;* see Kunter et al. 2002). Please note that the variables race/ethnicity and free or reduced price lunch that are often used in US samples are no meaningful categories in Germany.

### Analytic Strategy

#### Measurement invariance testing

Structural equation modeling (SEM) in Mplus 8 (Muthén and Muthén 1998–2017) was applied with standard errors corrected for the clustered data structure by the Mplus command “type = complex”. In order to meaningfully compare latent means between female and male students, (partial) scalar invariance of motivation and mindsets measures had to be established first. Measurement invariance testing comprises of a series of more and more restrictive models. More precisely, in a configural invariance model, only the factor structure is supposed to be invariant across male and female students, in a metric invariance model factor loadings are additionally set to be invariant, and in the most restrictive scalar invariance model also the intercepts are set to be invariant across female and male students (e.g., Putnick and Bornstein 2016). If a non-significant χ² difference test and Δ CFI < 0.01 (Cheung and Rensvold 2002) indicated that the model fit of the more restrictive model was not statistically significantly worse than the fit of the less restrictive model, measurement invariance was assumed. Additionally, the results from χ²-tests, the CFI, the RMSEA with its 90% confidence interval, and the SRMR are reported with the following cut-off criteria as indicating a good (acceptable) model fit: CFI ≥ 0.95 (0.90), RMSEA ≤ 0.06 (0.08), SRMR ≤ 0.08 (Hu and Bentler 1999).

Since fixed mindsets were measured by only two items, its measurement model would not be identified. Thus students’ fixed mindsets could not be tested for measurement invariance across gender in individual models. Therefore, its measurement invariance was tested simultaneously with the measurement invariance of either intrinsic motivation or ability self-concept which were both assessed by four items. In these models, the two factor loadings of the mindset construct were constrained to be equal and the relation between the motivational construct and the mindset fixed to zero.

#### Multigroup analyses

Multigroup SEM with “type = complex” and students’ gender functioning as the grouping variable was used to test whether mindsets were differently predictive for female and male adolescents’ ability self-concept (H1a and 2a) and intrinsic motivation (H2a and 2b) separately in the two domains. Students’ mindsets, ability self-concept and intrinsic motivation were specified as latent variables. To control for the effects of student characteristics that might be confounded with students’ mindset in math and language arts, the control variables students’ prior achievement, competence test scores, language students spoke at home and number of books at home were added as manifest predictors in a second step. The Wald χ^2^-Test indicated whether the regression coefficients differed statistically significantly between the female and male adolescents. Also here, CFI, RMSEA, and SRMR served as fit indices.

## Results

### Measurement Invariance Testing

Scalar invariance across gender was established for students’ ability self-concept, mindset, and intrinsic motivation in math (see Table S2 in the online supplement), and for students’ ability self-concept and mindset in language arts (see Table S3 in the online supplement). For intrinsic motivation in language arts, Item 1 and Item 4 were allowed to covary in order to have a satisfactory model fit. Moreover, in order to achieve partial scalar invariance, the intercept of Item 4 was freed. Since more than half of the intrinsic motivation items thus were invariant, partial scalar invariance was considered to be given (Putnick and Bornstein 2016).

### Descriptive Statistics

Means and standard deviations are presented in Table 1. As in previous studies with students of this age (e.g., Gunderson et al. 2017), students reported stronger fixed mindsets in math than in language arts, *T*(783) = 4.20, *p* < 0.001, *d* = 0.13.

**Table 1:** Means, standard deviations, and latent correlations

	*M* (SD)	1	2	3	4	5	6	7	8	9	10	11	12
1) Fixed mindset Math	3.00 (1.52)		−0.26**	−0.22***	0.05	0.02	0.81***	0.12	0.07	−0.10	0.07	−0.03	0.12*
2) ASC Math	3.15 (0.95)	−0.02		0.80***	−0.61***	0.44***	−0.02	−0.06	−0.16**	−0.22***	−0.04	0.06	0.13**
3) IM Math	2.67 (1.09)	−0.01	0.75***		−0.47***	0.33***	−0.03	−0.15*	0.02	−0.15*	−0.08	0.05	0.09
4) Grades Math	3.19 (1.03)	−0.07	−0.53***	−0.39***		−0.37***	−0.07	0.03	0.11**	0.39***	−0.05	0.01	−0.14*
5) Math competence	18.55 (8.26)	0.12*	0.37***	0.25***	−0.31***		−0.00	0.04	−0.07	−0.31***	0.20**	0.06	0.24***
6) Fixed mindset LA	2.80 (1.44)	0.77***	0.03	0.02	0.05	0.01		−0.06	−0.00	−0.01	−0.08	−0.00	0.13*
7) ASC LA	3.42 (0.73)	0.01	−0.21**	−0.15*	0.12**	−0.14*	−0.04		0.64***	−0.43***	0.17**	−0.09	0.28***
8) IM LA	2.89 (0.91)	0.06	−0.16**	0.17**	0.10	−0.16*	0.02	0.64***		−0.23***	−0.02	−0.01	0.13*
9) Grades LA	3.10 (0.89)	−0.02	−0.04	−0.04	0.35***	−0.16**	0.05	−0.41***	−0.20***		−0.28***	0.10	−0.27***
10) Reading comprehension	9.72 (5.45)	−0.06	0.08	0.00	−0.12*	0.31***	−0.05	0.07	−0.09	−0.24***		−0.21***	0.23***
11) Language at home	0.17 (0.38)	0.04	−0.09	−0.09	0.05	−0.06	−0.04	−0.06	0.02	0.22***	−0.30***		−0.15**
12) Number books	2.88 (1.35)	0.01	0.12	0.05	−0.17**	0.21***	−0.05	0.07	−0.00	−0.29***	0.20***	−0.22***	

ASC = ability self-concept. IM = intrinsic motivation. LA = German language arts. Fixed mindsets were assessed on a scale ranging from 1 to 7. ASC and IM were assessed on a scale ranging from 1 to 5. Grades indicating students’ prior achievement ranged from 1 = *excellent* to 6 = *insufficient*. Math competence test scores could theoretically range from 0 to 50, reading comprehension test scores from −23 to 46. Language at home indicated the language students spoke at home (0 = *German*, 1 = *other language*). Latent correlations for female students (*n* = 423) are presented above the diagonal, latent correlations for male students (*n* = 446) below the diagonal

*p < 0.05; **p <0.01; ***p < 0.001

Next, latent means of motivation and mindsets between female and male adolescents in math and language arts, respectively, were compared. Positive values indicate higher scores for male than female students, negative values vice versa. The results indicated that male students reported higher ability self-concepts (*d* = 0.41, *p* < 0.001) and intrinsic motivation (*d* = 0.24, *p* = 0.001) in math than female students. These effect sizes are in line with those found in earlier studies for this age group (e.g., Skaalvik and Skaalvik 2004). Female students in turn reported higher ability self-concepts (*d* = −0.52, *p* < 0.001) and intrinsic motivation (*d* = −0.49, *p* < 0.001) in language arts. Again the effect sizes fall into the range of effect sizes reported in prior research (e.g., Heyder et al. 2017). Female students did not report more fixed mindsets in math than male students (*d* = 0.06, *p* = 0.476), neither did male students for language arts (*d* = 0.09, *p* = 0.212).

Latent bivariate correlations (see Table 1) indicated higher ability self-concepts and intrinsic motivation for students with high prior achievement. Furthermore, female students’ fixed mindsets were negatively correlated with their ability self-concept and intrinsic motivation in math.

### Gender-Specific Effects of Fixed Mindsets in Math

The fit indices for the SEMs in math without and with control variables indicated very good model fits (see Table 2). The following results are from the models with control variables. Please note that the findings did not differ substantially between both models and that both are presented in Table 2.

**Table 2:** Results of the multigroup structural equation models predicting ability self-concept and intrinsic motivation in math

	Ability self-concept				Intrinsic motivation			
	*B* (SE) Female	*B* (SE) Male	*B* (SE) Female	*B* (SE) Male	*B* (SE) Female	*B* (SE) Male	*B* (SE) Female	*B* (SE) Male
Fixed Mindset	−0.25 (0.08)**	−0.01 (0.05)	−0.23 (0.07)**^a^	−0.07 (0.04)^a^	−0.26 (0.08)**	−0.01 (0.07)	−0.23 (0.07)**^b^	−0.07 (0.06)^b^
Prior achievement			−0.46 (0.04)***	−0.43 (0.05)***			−0.49 (0.06)***	−0.43 (0.05)***
Math competence			0.03 (0.01)***	0.03 (0.01)***			0.03 (0.01)***	0.02 (0.01)**
Language at home			0.12 (0.10)	−0.13 (0.12)			0.11 (0.15)^c^	−0.24 (0.13)^c^
Number books			0.02 (0.03)	−0.03 (0.03)			0.00 (0.04)	−0.05 (0.06)
Model fit								
χ² (df)	38.05 (26)		92.30 (66)*		70.82 (26)***		144.82 (66)***	
CFI	0.995		0.991		0.978		0.968	
RMSEA [90% CI]	0.033 [0.000; 0.054]		0.031 [0.013; 0.045]		0.063 [0.046; 0.081]		0.054 [0.042; 0.065]	
SRMR	0.040		0.041		0.035		0.039	

*N* = 832. Unstandardized coefficients and standard errors in parentheses. Prior achievement ranged from 1 = *excellent* to 6 = *insufficient*. Identical superscripts indicate that the regression coefficients for female and male students differed significantly (*p* < 0.05)

*p < 0.05; **p < 0.01; ***p < 0.001

Multigroup analyses showed that a fixed mindset negatively predicted^2^ ability self-concept in math only among female but not male students. The Wald χ²-Test indicated that this difference was statistically significant, *χ*² (1) = 5.08, *p* = 0.024, supporting H1a. Consistent with prior work (e.g., Möller et al. 2009), students with low prior achievement and low math competence reported lower ability self-concepts.

Furthermore, a fixed mindset of math ability was a negative predictor of intrinsic motivation in math only for female but not for male students (see Table 2). This gender difference was statistically significant as well, Wald Test: *χ*² (1) = 4.43, *p* = 0.035, supporting H1b. Moreover, students with low prior achievement and low math competence reported statistically significantly lower intrinsic motivation as found in prior research (e.g., Heyder et al. 2020).

### Gender-Specific Effects of Fixed Mindsets in Language Arts

Also for language arts, the fit indices indicated a very good model fit for the models without and with control variables (see Table 3). Again, all results are presented in the table and the results in the text refer to the models with control variables. Contrary to H2a, a fixed mindset in language arts was not significantly related to male students’ ability self-concept in language arts, and there was no significant gender difference, Wald Test: *χ*² (1) = 0.29, *p* = 0.589. Low-achieving students reported more negative ability self-concepts in language arts, which is consistent with prior work (e.g., Möller et al. 2009).

**Table 3:** Results of the multigroup structural equation models predicting ability self-concept and intrinsic motivation in language arts

	Ability self-concept				Intrinsic motivation			
	*B* (SE) Female	*B* (SE) Male	*B* (SE) Female	*B* (SE) Male	*B* (SE) Female	*B* (SE) Male	*B* (SE) Female	*B* (SE) Male
Fixed Mindset	−0.04 (0.04)	−0.03 (0.04)	−0.06 (0.04)	−0.03 (0.04)	−0.02 (0.05)	−0.01 (0.05)	−0.02 (0.05)	−0.01 (0.05)
Prior achievement			−0.28 (0.04)***	−0.30 (0.05)***			−0.19 (0.06)***	−0.21 (0.06)***
Reading compr.			0.00 (0.01)	−0.00 (0.01)			−0.02 (0.01)	−0.02 (0.01)
Language at home			−0.02 (0.08)	0.03 (0.08)			0.03 (0.10)	0.02 (0.11)
Number books			0.09 (0.03)***^a^	−0.02 (0.03)^a^			0.06 (0.03)	−0.01 (0.04)
Model fit								
χ² (df)	31.58 (26)		84.12 (66)		29.35 (26)		98.87 (62)	
CFI	0.997		0.991		0.996		0.982	
RMSEA [90% CI]	0.022 [0.000; 0.046]		0.026 [0.000; 0.041]		0.028 [.000; .052]		0.038 [.023; .051]	
SRMR	0.032		0.033		0.030		0.034	

*N* = 828. Unstandardized coefficients and standard errors in parentheses. Reading compr. = reading comprehension. Prior achievement ranged from 1 = *excellent* to 6 = *insufficient*. Identical superscripts indicate that the regression coefficients for female and male students differ significantly (*p* < 0.05)

**p < 0.01; ***p < 0.001

Contrary to H2b, a fixed mindset in language arts was neither significantly related to male students’ intrinsic motivation in German (see Table 3) and there was no significant gender difference, Wald Test: *χ*² (1) = 0.01, *p* = 0.916. Only students’ prior achievement was a significant predictor of students’ intrinsic motivation in language arts in that low-achieving students reported lower intrinsic motivation (e.g., Gniewosz et al. 2015).

## Discussion

In spite of various initiatives promoting gender equality, women are still overrepresented in the verbal domain and underrepresented in math and science (e.g., National Science Board 2018). Two important motivational predictors of students’ choices are their domain-specific ability self-concept and intrinsic motivation (Wang and Degol 2013) which are amongst others shaped by students’ gender stereotypes and mindsets (Eccles [Parsons] et al. 1983). Against this background, this study tested the relation between having a domain-specific fixed mindset and students’ ability self-concept and intrinsic motivation in math and language arts in male and female adolescents. In line with the hypotheses, the results showed that having a fixed mindset of math ability was associated with a lower ability self-concept and lower intrinsic motivation in math only for female students, but not for male students. Male students’ ability self-concept and intrinsic motivation in math were unrelated to their mindset in this domain. For the verbal domain, students’ mindset of language arts ability was found to be unrelated to their ability self-concept and intrinsic motivation in both male and female students.

### Role of Female and Male Students’ Mindsets in Math

This study’s results suggest that having a fixed mindset of math ability is a burden for female students’ motivation in math. This finding supports prior research that found similar effects on female students’ expectations of success (Degol et al. 2018), sense of belonging (Rattan et al. 2018), and achievement in math (Good et al. 2003). The study contributes to the knowledge in this field by showing that female students’ fixed mindset in math was associated with not only a lower ability self-concept, but also with lower intrinsic motivation. This is important because the combination of having a low ability self-concept and lower intrinsic motivation lowers the chances of pursuing a math-related career, above and beyond the individual effects of the constructs (Lauermann et al. 2017). Since students’ competencies, prior achievement, language spoken at home and number of books were statistically controlled for, these variables have unlikely confounded these results.

With regard to boys and men in math, prior research has found inconsistent results on whether mindsets do (Degol et al. 2018 for values; Seo et al. 2019 for math self-concept) or do not matter (Degol et al. 2018 for expectancy beliefs; Good et al. 2003 for test scores; Rattan et al. 2018 for sense of belonging). This study found that having a fixed mindset was unrelated to male students’ ability self-concept and intrinsic motivation. These findings suggest that knowing to belong to the social group that is ascribed a rather high math ability might buffer the negative effects of believing in an “innate” ability required to succeed. Possible explanations for the diverging findings in this field might be found in the different study designs and samples. To illustrate, whereas Degol et al. (2018) assessed mindset and motivation simultaneously and controlled for prior achievement and students’ socio-economic and cultural background (as done here), Seo et al. (2019) studied the relation between students’ mindset and math achievement in Grade 10, self-concept and math achievement in Grade 12, and STEM career expectancy beliefs and STEM career attainment two years later each (each construct measured only once except math achievement), and tested whether relations differ between male and female students from different racial/ethnic groups. Degol et al. (2018) relied on math course grades, Seo et al. (2019) on standardized math tests, and in this study both, prior grades and current competencies, were controlled for. Whereas this study focused also on tenth graders, Degol et al. (2018) studied students attending Grades 9–12. Both Degol et al. (2018) and Seo et al. (2019) used samples from the US whereas here German students were studied. Future research is needed in order to investigate whether cultural, developmental, or methodological characteristics are the reason for the partly inconsistent findings.

The results of this study as such do not allow conclusions about the causal effects of students’ mindsets. However, together with earlier work they suggest to pay close attention to implicit and explicit messages given to (female) students by teachers (e.g., Heyder et al. 2020) or parents (e.g., Moorman and Pomerantz 2010) about the nature of math ability in order to keep female adolescents in STEM courses and increase the share of women in STEM occupations later on.

### Role of Female and Male Students’ Mindsets in Language Arts

Whereas prior research on gender-specific effects of students’ mindsets so far focused on math and the US, this study analyzed the effects in a German sample in language arts as well. Female students’ mindset in language arts was unrelated to their motivation, mirroring the findings for male students in math. In contrast to the findings for female students in math, however, the analyses suggest that a fixed mindset in language arts is *not* negatively related to male students’ ability self-concept and the intrinsic motivation, although male students are stereotyped as having less “innate” verbal talent (e.g., Steffens and Jelenec 2011). There are two theoretical explanations for this unexpected finding. A first explanation might be found in results from this study and other studies (e.g., Gunderson et al. 2017; for similar findings on teachers, see Heyder et al. 2020) suggesting that students do not believe as much in an “innate” ability required to be successful in language arts as they do so for math. Thus, even if a male student believes in an “innate” language arts ability and is ascribed less of this ability because of his gender, he might still consider this ability as less important for being successful than other characteristics such as the instruction or the teacher, protecting his ability self-concept and intrinsic motivation. A second explanation could be derived from recent studies indicating that already from childhood on, intellectual brilliance, that is domain-unspecific superior intellectual functioning, is more strongly associated with male than female persons (e.g., Bian et al 2018). Such brilliance-male stereotype might compensate the negative effects expected from fixed mindsets and language-female stereotypes for male students and thus explain, why fixed mindsets were unrelated to male students’ motivation here. Since this is the first study on the relation between mindsets and motivation in language arts, further research is needed to shed more light on the mechanisms behind the relations in language arts revealed here.

### Limitations and Future Research Directions

Limitations of this study first concern the cross-sectional study design that does not allow testing the direction of effect or any causal conclusions as already mentioned before. Thus experimental research or longitudinal cross-lagged models are needed in order to gain more insights into the developmental dynamics of students’ mindsets, motivation, and achievement, and test whether the relations revealed here and in earlier related research (i.e., Degol et al. 2018; Seo et al. 2019) are in fact causal. Second, research indicated that math-male gender stereotypes are still prevalent (e.g., Chestnut and Markman 2018) but they were not assessed in the FA(IR)BULOUS project and thus not included in this study. Theoretically it seems plausible that fixed mindset effects become larger, the stronger female students believe in women having less “innate” math ability than men. However, this plausible moderation by students’ gender stereotype endorsement was beyond the scope of this study and therefore remains an interesting task for future research.

Third, this study focused on low- and medium track students that appear prone to detrimental fixed mindset effects due to derogatory beliefs about low- and medium track students (e.g., Knigge and Hannover 2011). Thus, it is not know yet whether the results for students attending academic tracks that signal a higher level of ability are similar. Furthermore, members of other social groups might be vulnerable as well, such as students from low socioeconomic backgrounds (Sisk et al. 2018) or with low achievement (e.g., Yeager et al. 2019, for teacher mindset effects, see Heyder et al. 2020). With regard to language arts, a fixed mindset might be particularly detrimental to students with a mother tongue that is not the language of instruction in school. In future studies, it thus would seem fruitful to consider additional student characteristics or their intersectionality to gain a more comprehensive picture of the role of students’ domain-specific mindsets for their motivation (see also Seo et al. 2019). Moreover, it would be promising to assess not only students’ ability self-concept and intrinsic motivation in language arts, but also their mid-term educational and occupational choices related to the verbal domain as already done for math (e.g., Seo et al. 2019).

## Conclusion

The lower math ability self-concept and intrinsic motivation in math of female than male adolescents are two important precursors of women’s underrepresentation in prestigious STEM occupations. Prior solely US based research suggested that a fixed mindset of math ability may represent an additional burden for female adolescents in math, but findings vary between constructs and measures. Whether this potential burden tackles both female adolescents’ ability self-concept and intrinsic motivation in math and holds for samples from outside the US, and whether opposite relations exist in academic domains dominated by women has been unclear. To address this research gap, this study analyzed the relation between adolescents’ mindset, ability self-concept, and intrinsic motivation in two gender-stereotyped and opposed domains, math and German language art, in Germany. Multigroup SEM showed that having a fixed mindset was negatively related to female adolescents’ ability self-concept and intrinsic motivation in math, but not to male adolescents’, and unrelated to adolescents’ motivation in language arts. These findings suggest that having a fixed mindset of math ability represents an additional burden for female adolescents to persist and succeed in math. Since the gender-specific effects of students’ mindsets in different academic domains have not been studied systematically, there is need for experimental studies on the effects of mindsets that include the domain as well as students’ gender as potential moderator variables and shed more light on the mechanisms behind the findings.

## Supplementary information

Supplementary Material

## Footnotes

1 Schools of the academic track (Gymnasium) that make up around a third of the student population were not part of the FA(IR)BULOUS project.

2 The term predicted is used here and in the following in order to make transparent that a directional relation was specified and not a covariance. Please note that the models are still based on cross-sectional data that do not allow testing the direction of an effect.
