# Supplementary material for: Only a Burden for Females in Math? Gender and Domain Differences in the Relation Between Adolescents’ Fixed Mindsets and Motivation
Source: J Youth Adolesc. 2020 Nov 10;50(1):177–88. doi: 10.1007/s10964-020-01345-4 (PMC7815566; doi:10.1007/s10964-020-01345-4)
Supplement: Supplementary file 1 — Supplementary Material [file 10964_2020_1345_MOESM1_ESM.pdf]

Supplementary Material

Heyder, A., Weidinger, A.F., & Steinmayr, R. (in press). Only a burden for females in math? Gender and domain differences in the relation between adolescents' fixed mindsets and motivation. *Journal of Youth and Adolescence.*

Table S1

Comparison of FA(IR)BULOUS Sample and Population Characteristics

Student variables	Analytical Sample	Population
Female students	48.2%	47.3%
Comprehensive school (Gesamtschule)	30.1%	32.2%
Intermediate track (Realschule)	53.0%	46.9%
Lowest track (Hauptschule)	16.9%	20.9%
German spoken at home	82.0%	73.4%

Note. The student population data was provided by the Ministry for School and Further Education North Rhine-Westphalia (MSW, 2017).

Table S2

Measurement Invariance Tests for Female vs. Male Adolescents' Ability Self-Concept, Intrinsic Motivation and Mindset in Math

		Model fit						Model comparison				
		X^2	*df*	*p*	CFI	RMSEA [90% CI]	SRMR	CM	Δ X^2	Δ *df*	*p*	Δ CFI
ASC and fixed mindset math	(1) Configural	38.57	18	.003	.991	.051 [.029; .074]	.072					
	(2) Metric	45.16	23	.004	.990	.047 [.026; .068]	.073	(1)	1.36	5	.929	-.001
	(3) Scalar	49.56	27	.005	.990	.044 [.024; .063]	.074	(2)	4.74	4	.315	0
IM and fixed mindset math	(1) Configural	62.47	18	<.001	.979	.076 [.056; .097]	.060					
	(2) Metric	71.76	23	<.001	.976	.070 [.052; .089]	.060	(1)	0.54	5	.994	-.003
	(3) Scalar	78.92	27	<.001	.975	.067 [.050; .084]	.061	(2)	7.10	4	.131	-.001

Note. ASC = ability self-concept. IM = intrinsic motivation. Because student's mindset had only two indicators, it was tested for measurement invariance simultaneously with IM and ASC. The covariance between the constructs was fixed to zero. CM = comparison model.

Table S3

Measurement Invariance Tests for Female vs. Male Adolescents' Ability Self-Concept, Intrinsic Motivation and Mindset in Language Arts

		Model fit						Model comparison				
		X^2	*df*	*p*	CFI	RMSEA [90% CI]	SRMR	CM	Δ X^2	Δ *df*	*p*	Δ CFI
ASC and fixed mindset LA	(1) Configural	24.26	18	.149	.996	.028 [.000; .055]	.027					
	(2) Metric	27.70	23	.228	.997	.022 [.022; .047]	.030	(1)	1.11	5	.953	.001
	(3) Scalar	31.60	27	.247	.997	.020 [.000; .044]	.034	(2)	3.89	4	.422	0
IM and fixed mindset LA	(1) Configural	24.23	16	.085	.996	.035 [.000; .061]	.025					
	(2) Metric	29.83	21	.095	.996	.031 [.000; .055]	.030	(1)	4.26	5	.513	0
	(3) Scalar	47.63	25	.004	.989	.046 [.025; .065]	.038	(2)	16.68	4	.002	-.007
	(4) Partial scalar[a]	29.25	24	.211	.997	.023 [.000; .047]	.030	(2)	0.58	3	.996	.001

Note. ASC = ability self-concept. IM = intrinsic motivation. LA = German language arts. The first and fourth IM items were allowed to correlate. Because student's mindset had only two indicators, it was tested for measurement invariance simultaneously with IM and ASC. The covariance between the constructs was fixed to zero. CM = comparison model.

[a] The intercept of the fourth IM item was freed.
